# Vitamin D levels in children and adolescents with chronic tic disorders: a multicentre study

**DOI:** 10.1007/s00787-021-01757-y

**Published:** 2021-04-13

**Authors:** Molly Bond, Natalie Moll, Alicia Rosello, Rod Bond, Jaana Schnell, Bianka Burger, Pieter J. Hoekstra, Andrea Dietrich, Anette Schrag, Eva Kocovska, Davide Martino, Norbert Mueller, Markus Schwarz, Ute-Christiane Meier, the EMTICS Collaborative Group, Julie E. Bruun, Judy Grejsen, Christine L. Ommundsen, Mette Rubæk, Stephanie Enghardt, Stefanie Bokemeyer, Christiane Driedger-Garbe, Cornelia Reichert, Jenny Schmalfeld, Thomas Duffield, Franciska Gergye, Margit Kovacs, Reka Vidomusz, Miri Carmel, Silvana Fennig, Ella Gev, Nathan Keller, Elena Michaelovsky, Matan Nahon, Chen Regev, Tomer Simcha, Gill Smollan, Avi Weizman, Giuseppe Gagliardi, Marco Tallon, Paolo Roazzi, Els van den Ban, Sebastian F. T. M. de Bruijn, Nicole Driessen, Andreas Lamerz, Marieke Messchendorp, Judith J. G. Rath, Nadine Schalk Deborah Sival, Noor Tromp, Frank Visscher, Stichting Gilles de la Tourettes, Maria Teresa Cáceres, Fátima Carrillo, Pilar Gómez-Garre, Laura Vargas, Maria Gariup, Sara Stöber, Alan Apter, Valentina Baglioni, Juliane Ball, Noa Benaroya-Milshtein, Benjamin Bodmer, Molly Bond, Emese Bognar, Bianka Burger, Judith Buse, Francesco Cardona, Marta Correa Vela, Andrea Dietrich, Nanette M. Debes, Maria Cristina Ferro, Carolin Fremer, Blanca Garcia-Delgar, Mariangela Gulisano, Annelieke Hagen, Julie Hagstrøm, Tammy J. Hedderly, Isobel Heyman, Pieter J. Hoekstra, Chaim Huyser, Marcos Madruga-Garrido, Anna Marotta, Davide Martino, Ute-Christiane Meier, Pablo Mir, Natalie Moll, Astrid Morer, Norbert Mueller, Kirsten Müller-Vahl, Alexander Münchau, Peter Nagy, Valeria Neri, Thaïra J. C. Openneer, Alessandra Pellico, Ángela Periañez Vasco, Kerstin J. Plessen, Cesare Porcelli, Marina Redondo, Renata Rizzo, Veit Roessner, Daphna Ruhrman, Jaana M. L. Schnell, Anette Schrag, Marcus J. Schwarz, Paola Rosaria Silvestri, Liselotte Skov, Tamar Steinberg, Friederike Tagwerker Gloor, Zsanett Tarnok, Jennifer Tübing, Victoria L. Turner, Susanne Walitza, Elif Weidinger, Martin L. Woods

**Affiliations:** 1 Blizard Institute, Barts and The London School of Medicine and Dentistry, Queen Mary University of London, 4 Newark Street, London, E1 2AT UK; 2 Institute of Laboratory Medicine, University Hospital, LMU Munich, Munich, Germany; 3 Statistics, Modelling and Economic Department, National Infection Service, PHE, London, UK; 4 Department of Infectious Disease Epidemiology, London School of Hygiene and Tropical Medicine, London, UK; 5 University of Sussex, Brighton, UK; 6 Department of Child and Adolescent Psychiatry, University Medical Center Groningen, University of Groningen, Groningen, The Netherlands; 7 Department of Clinical Neuroscience, UCL Institute of Neurology, University College London, London, UK; 8 Department of Psychiatry and Psychotherapy, University Hospital, LMU Munich, Munich, Germany; 9 Institute of Psychiatry, Psychology and Neuroscience, King’s College London, London, UK; 10 Ludwig-Maximilians-University Munich, Munich, Germany; 11 Department of Clinical Neurosciences, Cumming School of Medicine and Hotchkiss Brain Institute, University of Calgary, Calgary, AB Canada

**Keywords:** Tic disorder, Vitamin D, ADHD, OCD, Tourette, Pediatrics

## Abstract

This study investigated whether vitamin D is associated with the presence or severity of chronic tic disorders and their psychiatric comorbidities. This cross-sectional study compared serum 25-hydroxyvitamin D [25(OH)D] (ng/ml) levels among three groups: children and adolescents (3–16 years) with CTD (*n* = 327); first-degree relatives (3–10 years) of individuals with CTD who were assessed for a period of up to 7 years for possible onset of tics and developed tics within this period (*n* = 31); and first-degree relatives who did not develop tics and were ≥ 10 years old at their last assessment (*n* = 93). The relationship between 25(OH)D and the presence and severity of tics, as well as comorbid obsessive–compulsive disorder (OCD) and attention-deficit/hyperactivity disorder (ADHD), were analysed controlling for age, sex, season, centre, latitude, family relatedness, and comorbidities. When comparing the CTD cohort to the unaffected cohort, the observed result was contrary to the one expected: a 10 ng/ml increase in 25(OH)D was associated with higher odds of having CTD (OR 2.08, 95% CI 1.27–3.42, *p* < 0.01). There was no association between 25(OH)D and tic severity. However, a 10 ng/ml increase in 25(OH)D was associated with lower odds of having comorbid ADHD within the CTD cohort (OR 0.55, 95% CI 0.36–0.84, *p* = 0.01) and was inversely associated with ADHD symptom severity (*β* = − 2.52, 95% CI − 4.16–0.88, *p* < 0.01). In conclusion, lower vitamin D levels were not associated with a higher presence or severity of tics but were associated with the presence and severity of comorbid ADHD in children and adolescents with CTD.

## Introduction

Chronic tic disorders (CTD) are neurodevelopmental conditions of which there are three types: chronic motor tic disorder, chronic vocal tic disorder, and Tourette Syndrome (TS) [3]. TS is characterised by the combined presence of multiple motor tics and at least one vocal tic and has an estimated prevalence of 0.3–0.9% [2, 19, 36]. A large proportion of individuals with CTD (~ 40–80%) have associated neuropsychiatric comorbidities; obsessive–compulsive disorder (OCD) and attention-deficit/hyperactivity disorder (ADHD) are among the most common  [36]. Tics and their associated neuropsychiatric comorbidities, and ADHD in particular, have been shown to have a negative impact on quality of life and socioeconomic status [1, 8, 9, 17].

The aetiology of CTD is not fully understood. Estimates of TS heritability range from 0.25 to 0.77 [29, 44], suggesting that environmental factors also play a substantial role. CTD, like most other neuropsychiatric diseases, is a polygenic disorder [38]. Susceptibility genes so far identified include genes involved in multiple neurotransmitter systems, as well as the immune system [27, 42]. Environmental factors include psychosocial stress, maternal smoking during pregnancy, and potentially infectious pathogens, although the evidence remains inconclusive [15].

One environmental factor of interest is vitamin D, a neurosteroid hormone, which plays an important role in skeletal health, as well as in neural and immune functioning [10]. Vitamin D is a fat-soluble vitamin that is naturally present in a few foods and mainly produced in the skin when it is exposed to sunlight (UVB radiation). Its synthesis is therefore greatly influenced by season, latitude, air pollution, skin pigmentation, sunscreen use and aging, as well as by genetic factors, altered absorption/metabolism, and medication [20]. Vitamin D influences a large number of biological pathways; its receptors and activating enzyme (1α-hydroxylase) are widespread throughout the human brain, and it is thought to have neurotropic and neuroprotective effects, influencing on neurotransmission, neuroplasticity, and neuroinflammation [10, 20]. This may explain why hypovitaminosis D has been linked to several neuropsychiatric and neurological diseases such as mood disorders, schizophrenia, autism, multiple sclerosis, Parkinson’s disease, and Alzheimer’s disease [10, 20].

Theoretically, there are a number of ways in which vitamin D could be associated with CTD. Dopaminergic dysfunction is widely considered the pathological endpoint of CTD [23], though preceding pathogenic pathways are poorly understood. Vitamin D regulates gene expression of the rate-limiting enzyme, tyrosine hydroxylase, which modulates the production of dopamine, noradrenaline, and epinephrine [7]. Therefore, deficiency in vitamin D could contribute to dopaminergic dysfunction in CTD. Another possibility is that vitamin D is associated with raised inflammatory markers that have been reported in CTD [28]. Vitamin D has a largely anti-inflammatory effect and impacts on cellular und humoral immune responses [20]. Thus, it is plausible that low vitamin D contributes to inflammation in CTD or, conversely, that an overactive immune system in CTD leads to increased consumption of vitamin D, and thus lower levels (“reverse causation”) [20]. Vitamin D is also known to be important in brain development [10], and abnormalities in the maturation and structure of the brain are thought to be involved in the pathogenesis of CTD [13]. As the pathophysiology of CTD is not yet fully understood and the effects of vitamin D are multiple throughout the body, it is difficult to pin-point a single possible underlying mechanism, though there are a number of ways in which the two could plausibly be linked.

Evidence from one case–control study (*n* = 179) and a follow-up supplementation trial (*n* = 36) in Chinese children suggested vitamin D may be associated with tic disorders [24, 25]. These two studies reported lower levels of 25-hydroxyvitamin D (25[OH]D) in children with tics compared to healthy controls; a negative correlation between tic severity and 25(OH)D levels (*r* = − 0.32); and observed improvements in tic severity following 3-month supplementation in people with CTD who were insufficient or deficient in 25(OH)D at study entry (average reduction in severity score from [mean ± SD] 23 ± 5 to 13 ± 7, *t* = 10.15) [25]. OCD and ADHD, the most common comorbidities in CTD, have also been linked to lower levels of vitamin D [6, 21]. However, the result has been inconsistent [26, 43] and the salience of these observations remains to be understood.

Our study is part of the European Multicentre Tics in Children Studies (EMTICS) [39], a large prospective European multicentre study of children and adolescents with CTD and first-degree relatives of individuals with CTD who themselves did not have tics at study entry and were followed up for a maximum of 7 years to assess the possible onset of tics [39]. We hypothesised that: A. 25(OH)D is lower in individuals with CTD (CTD cohort) compared to first-degree relatives of people with CTD but who themselves do not have tics (unaffected cohort); B. 25(OH)D is lower in first-degree relatives who developed tic disorders (tic onset cohort) compared to those who did not develop tics (unaffected cohort) within the follow-up period; C. lower 25(OH)D is associated with greater tic severity in individuals with CTD; D. lower 25(OH)D is associated with greater presence and severity of comorbid OCD and ADHD in individuals with CTD. This is the largest study to date to assess 25(OH)D status in children and adolescents with CTD. It is unique in its assessment of 25(OH)D in first-degree relatives at risk of developing tics; and it is the first study to investigate an association between 25(OH)D levels and the severity of psychiatric comorbidities in CTD.

## Methods

### Participants

Participants were from the European Multicentre Tics in Children Studies (EMTICS) [39], a prospective longitudinal observational study involving 16 centres located in nine countries across Europe and in Israel. The structure of the study was previously described in detail (see [39]). EMTICS recruited two cohorts: the COURSE study, 703 children and adolescents (3–16 years) with an established diagnosis of CTD according to DSM-IV-TR (note that six participants enrolled in the study were removed as they did not have a CTD) [2]; and the ONSET study, an at-risk cohort comprising 259 first-degree relatives of children with CTD (3–10 years), without tics (note that one of the 260 participants enrolled in the study was recently removed due to tics prior to study entry). The at-risk cohort was followed up for 3 years to assess the onset of tics according to regular study protocol, during which time 61 tic onsets were identified. All unaffected children were reassessed via a brief telephone interview after the study ended, which identified seven additional confirmed tic onsets (*n* = 68 in total) in 2019/2020; 36 children could not be recontacted.

This study makes comparisons between participants with tic disorders and an unaffected group. The mean age of tic onset in the EMTICS ONSET cohort was 7.93 years old (SD 2.00, range 3.52–13). Therefore, there was a risk that children in the unaffected cohort might develop tics beyond the study period. To mitigate this risk, we only included participants who did not develop tics and were at least 10 years of age at the time of the extended follow-up reassessment or, for those that could not be reassessed, at least 10 years of age at the time of the last visit during the regular study term.

This cross-sectional study represented a sub-sample of the EMTICS cohorts based on available serum samples for 25(OH)D analysis. This included: baseline samples for 327 participants with CTD (CTD cohort); baseline samples for 93 participants who did not develop tics and were 10 years of age or older at the time of their last assessment (unaffected cohort); and samples taken at the time of tic onset for 31 participants who developed tics within the 3-year regular study period (tic onset cohort). (Note that no serum samples were taken during the telephone reassessment period and therefore the seven children who developed tics after the regular study period could not be included in the analyses of this study).

The regular study period took place from January 2013 to June 2018, and the final telephone reassessment concluded in May 2020. The study was approved by the Institutional Review Boards of the participating centres. Parents and their child(ren) provided written informed consent and assent according to the appropriate ethical regulations.

### Clinical assessment

An established diagnosis of CTD, OCD, and ADHD was confirmed by study clinicians according to DSM-IV-TR criteria [2]. Our main outcome measure for current tic severity was the Yale Global Tic Severity Scale (YGTSS) [22]. Study clinicians also rated the Clinical Global Impression Scale Severity (CGI) for tic severity during the previous week [14]. The Children’s Yale-Brown Obsessive–Compulsive Scale (CY-BOCS) was used to rate obsessive–compulsive symptom severity [12, 37]. The parent-reported Swanson, Nolan and Pelham-version IV rating scale (SNAP-IV) assessed ADHD symptom severity [41]. As SNAP-IV is parent-rated and not clinician assessed, we additionally used the DSM-IV-TR ADHD symptom count as a second measure of ADHD severity.

### Analysis of 25-hydroxyvitamin D (25(OH)D)

Serum samples were collected at study entry (i.e., at baseline) for the CTD cohort and for the unaffected cohort, whereas, for the tic onset cohort, we used serum samples that were taken at the time of tic onset. All samples were sent to the Department of Laboratory Medicine, Munich (LMU). The samples were stored at − 80 °C until the time of analysis. The level of 25(OH)D was measured in the ISO 15189 accredited lab on a DiaSorin Liaison analyser using chemiluminescent immunoassay technology. Serum samples were processed according to the manufacturer’s instructions. The range of the assay was 4 ng/ml (10 nmol/l) up to 150 ng/ml (375 nmol/l).

Hypovitaminosis-D was reported at levels under 20 ng/ml (50 nmol/l) in accordance with the US Endocrine Society [16], with levels ≤ 10 ng/ml (≤ 25 nmol/l) considered deficient and between 10 and 20 ng/ml (25–50 nmol/l) insufficient [16].

To correct for seasonal variation in 25(OH)D levels, we used a previously described method [30]. The following equation was used to predict 25(OH)D levels for any given day of the year for each cohort (*y* = 25(OH)D level and *t* = day of year, e.g., for January 1st, *t* = 0):

$$y_{i} = \beta_{0} + \beta_{1} \sin \left( {\frac{2\pi t}{{365}}} \right) + \beta_{2} \cos \left( {\frac{2\pi t}{{365}}} \right).$$

The difference between predicted 25(OH)D levels on a given day and the individual’s actual level gave a residual value of 25(OH)D controlling for season. In our analyses, we used multilevel models with site as a cluster variable to account for all differences between study centres, including, for example, ambient levels of vitamin D associated with living at different latitudes.

### Statistical analysis

Since this was a multicentre study involving siblings, participants were clustered both within centres and as families. Therefore, both centre and family relatedness were used as cluster variables in all multilevel models. Generalised linear mixed models were used to assess whether 25(OH)D levels were associated with: the odds of having a CTD compared to the unaffected cohort; tic onset compared to the unaffected cohort; and the presence of comorbid OCD or ADHD compared to those with CTD only. Multilevel models were used to investigate whether 25(OH)D levels were associated with tic severity (YGTSS and CGI) as well as the severity of comorbid OCD (CY-BOCS) and ADHD (SNAP-IV and DSM-IV-TR) symptoms. OCD and ADHD symptom severity scores were taken from the entire CTD cohort and not just those who met the criteria for each comorbid diagnosis.

Participants’ seasonally adjusted 25(OH)D level was the primary predictor variable in all analyses. To give a more clinically relevant outcome, adjusted 25(OH)D was divided by 10, so that one-unit change in the predictor variable was equal to 10 ng/ml 25(OH)D, as opposed to 1 ng/ml. As a secondary analysis, we ran all models with 25(OH)D as a binary predictor variable, with 25(OH)D insufficiency measured as ≤ 20 ng/ml and sufficiency as > 20 ng/ml. The average serum 25(OH)D was adjusted for by centre in order to disentangle contextual effects from person-level effects [35]. Age, sex, and the presence of a comorbidity (OCD and/or ADHD) were also entered as covariates.

As a sensitivity analysis, we re-analysed the generalised linear models in subsamples matched on age and sex [participants with CTD and unaffected (*n* = 93);  participants with tic onset and unaffected (*n* = 31); CTD participants with OCD and without OCD (*n* = 95); CTD participants with ADHD and without ADHD (*n* = 79)]. Subsamples were matched using propensity score matching in R [34].

The average predicted level of 25(OH)D is the same in May as it is at the end of November because these months fall at the halfway point between August (when levels are highest) and March (when they are lowest). Therefore, we added the average predicted 25(OH)D level for May/November to each individual’s residual value to estimate the proportion of participants in each cohort who would have insufficient levels of 25(OH)D for at least 6 months of the year (December–May).

## Results

### Study cohorts

Demographic and clinical characteristics of participants are shown in Table 1. The vast majority of the CTD cohort were diagnosed with TS (*n* = 298; 91.1%) and a minority with a motor CTD (*n* = 28; 8.6%) or vocal CTD (*n* = 1; 0.3%).

**Table 1:** Demographic and clinical characteristics

Characteristics	CTD (*n* = 327)	Tic onset (*n* = 31)	Unaffected (*n* = 93)
Age (years) (mean ± SD)	10.9 (± 2.72)	7.55 (± 1.83)	7.68 (± 1.76)
Sex *n* (%)			
Male	247 (75.5)	22 (71.0)	37 (39.8)
Female	80 (24.5)	9 (29.0)	56 (60.2)
Ethnicity *n* (%)			
White	301 (92.0)	25 (80.6)	80 (86.0)
Non-white	26 (8.0)	6 (19.4)	13 (14.0)
Tic Disorder *n* (%)			
Tourette Syndrome	298 (91.1)	8 (25.8)	
Chronic Motor Tic Disorder	28 (8.6)	7 (22.6)	
Chronic Vocal Tic Disorder	1 (0.3)		
Transient Tic Disorder		9 (29.0)	
Tic Disorder—NOS		7 (22.6)	
Comorbidities *n* (%)			
OCD	95 (29.1)		
ADHD	79 (24.2)	2 (6.5)	11 (11.8)
Seasonally Adjusted 25(OH)D *n* (%)			
Insufficient (10–20 ng/ml; 25–50 nmol/l)	89 (27.2)	8 (25.8)	35 (37.6)
Deficient (≤ 10 ng/ml; ≤ 25 nmol/l)	3 (0.9)	0 (0.0)	2 (2.2)
Median (interquartile range)	24.7 (19.2–29.8)	24.3 (19.6–33.7)	21.6 (17.0–27.3)

Unaffected cohort (did not develop tics by end of the study or reassessment period and ≥ 10 years old); non-white (mixed, Middle Eastern, North African, Asian or of unknown ancestry); NOS (tic disorder confirmed by type was ‘not otherwise specified’ by study clinicians)

*OCD* obsessive–compulsive disorder, *ADHD* attention deficit hyperactivity disorder

### Levels of 25(OH)D and prevalence of hypovitaminosis D in CTD, unaffected, and tic onset cohort

**Fig. 1:**
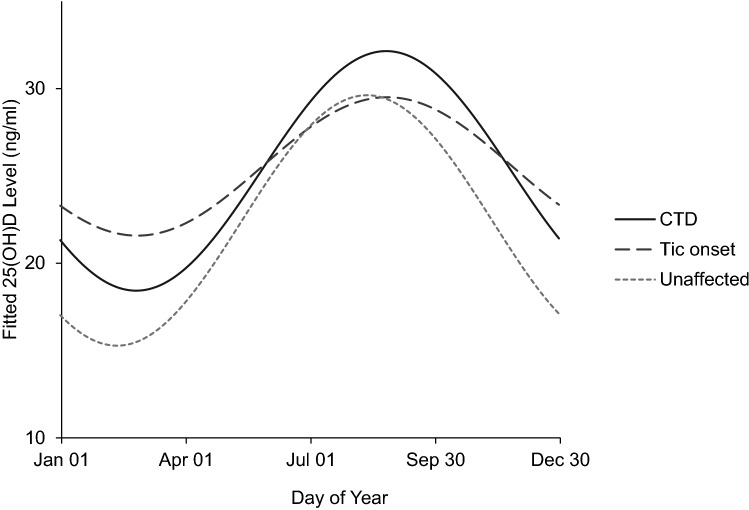
Fitted values from the sinusoidal regression model of vitamin D levels in terms of CTD/tic onset/unaffected and the day of the year on which the sample was taken (day 0 = January 1). This model was used to create a deseasonalised vitamin D level for each individual

Levels of 25(OH)D fluctuated according to season with the lowest levels in early spring and highest levels in late summer for all cohorts as shown in Fig. 1.

Contrary to our hypothesis, the unaffected cohort, who did not develop tics, had lower serum 25(OH)D levels than individuals in the CTD cohort and tic onset cohort. The sinusoidal regression model used to adjust for seasonal variation predicted hypovitaminosis D (25[OH]D < 20 ng/ml) in 43.7% of the unaffected cohort, 25.8% of the tic onset cohort, and 27.2% of the CTD cohort for 6 months of the year (December–May).

**Fig. 2:**
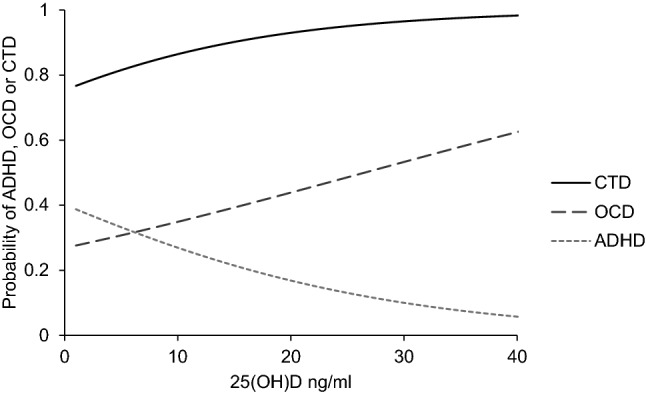
Estimated change in probability of having a CTD, OCD, or ADHD diagnosis as 25(OH)D (ng/ml) increases. As 25(OH)D increases, the probability of CTD and OCD increases, whereas for ADHD, the probability decreases

As shown in Table 2, a 10 ng/ml increase in 25(OH)D was associated with higher odds of having a CTD diagnosis when compared to the unaffected cohort (OR 2.08, 95% CI 1.27–3.42, *p* < 0.01). The change in probability of having CTD as 25(OH)D levels increase is shown in Fig. 2. However, this association was no longer significant when tested for in an age- and sex-matched subgroup (Table 4, Appendix 1).

**Table 2:** Effect of 25(OH)D on the presence of CTD, comorbid OCD, and ADHD

Dependent variable^a^	OR	95% CI	*p*
CTD	2.08	1.27–3.42	< 0.01*
Tic onset	1.73	0.91–3.29	0.10
CTD comorbid OCD	1.46	1.04–2.04	0.03*
CTD comorbid ADHD	0.55	0.36–0.84	0.01*

^a^The dependent variable is the presence of CTD, OCD, or ADHD and the primary predictor variable is adjusted 25(OH)D (1-unit change = 10 ng/ml), while sex, age, and comorbidity, other than that being tested, were also entered as covariates. The odds are calculated for 1-unit change (i.e., 10 ng/ml) in our predictor variable

*CTD* chronic tic disorder, *OCD* obsessive–compulsive disorder, *ADHD* attention-deficit hyperactivity disorder

*The significant difference with *p* < 0.05

There was no significant association between 25(OH)D levels and the first development of tics when comparing the tic onset cohort to the unaffected cohort (*p* = 0.10, Table 2).

### No association between 25(OH)D levels and the severity of tics in CTD cohort

As shown in Table 3, we found no significant association between 25(OH)D levels and tic severity as measured by both YGTSS (*p* = 0.84) and CGI (*p* = 0.91).

**Table 3:** Effect of 25(OH)D on Tic, OCD, and ADHD Symptom Severity

Dependent variable^a^	*β*	SE	95% CI	*p*
Tic severity				
YGTSS:Total	0.11	0.51	− 0.90 to 1.11	0.84
YGTSS Motor	0.03	0.29	− 0.54 to 0.61	0.91
YGTSS Vocal	0.08	0.34	− 0.60 to 0.76	0.82
CGI	− 0.01	0.06	− 0.13 to 0.11	0.91
OCD				
CYBOCs Total	0.55	0.49	− 0.42 to 1.51	0.27
CYBOCs Obsessions	0.25	0.26	− 0.26 to 0.77	0.33
CYBOCs Compulsions	0.30	0.34	− 0.37 to 0.98	0.37
ADHD				
DSM-IV-TR Total	− 1.02	0.33	− 1.67 to − 0.37	< 0.01*
DSM-IV-TR Inattentive	− 0.57	0.21	− 0.97 to − 0.16	0.01*
DSM-IV-TR Hyperactive/Impulsive	− 0.42	0.17	− 0.75 to − 0.09	0.01*
SNAP Total	− 2.52	0.83	− 4.16 to − 0.88	< 0.01*
SNAP Inattentive	− 1.75	0.47	− 2.68 to − 0.82	< 0.01*
SNAP Hyperactive/Impulsive	− 0.77	0.44	− 1.64 to 0.10	0.08

^a^The dependent variable is symptom severity. The primary predictor variable is adjusted 25(OH)D (1-unit change = 10 ng/ml), while age, sex, and comorbidity, other than that being tested, were also entered as covariates. *β* coefficient is calculated for 1-unit change (i.e., 10 ng/ml) in our predictor variable

*YGTSS* Yale Global Tic Severity Scale, *OCD* obsessive–compulsive disorder, *CY-BOCs* Children’s Yale-Brown Obsessive–Compulsive Scale, *ADHD* attention-deficit hyperactivity disorder, *DSM-IV-TR* diagnostic and statistical manual of mental disorders 4th ed., text revision, *SNAP-IV* Swanson, Nolan, and Pelham Questionnaire IV

*The significant difference with *p* < 0.05

### Association of 25(OH)D levels with comorbid ADHD and OCD in CTD cohort

*OCD*: as shown in Table 2 and Fig. 2, a 10 ng/ml increase in 25(OH)D was associated with higher odds of having a comorbid OCD diagnosis in the CTD cohort (OR 1.46, 95% CI 1.04 to 2.04, *p* = 0.03). However, we found no significant association between 25(OH)D and OCD symptom severity among the CTD cohort (*p* > 0.05) (Table 3).

**Fig. 3:**
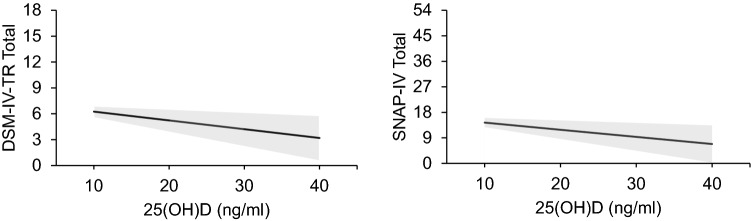
The graph on the left shows that as 25(OH)D increases, severity of ADHD as measured by DSM-IV-TR decreases. The graph on the right shows that as 25(OH)D increases, SNAP-IV symptom count decreases

*ADHD*: A 10 ng/ml increase in 25(OH)D was associated with lower odds of having an ADHD diagnosis in the CTD cohort (OR 0.55, 95% CI 0.36–0.84, *p* = 0.01) (Table 2 and Fig. 2). As shown in Fig. 3, there was also an inverse association between an increase of 10 ng/ml of 25(OH)D and SNAP-IV parent-rated ADHD symptom severity (*β* = − 2.52, S.E. = 0.83, *p* < 0.01), as well as for DSM-IV-TR ADHD total symptom count (*β* = − 1.02, S.E. = 0.33, *p* < 0.01) (Table 3). The association existed for the ADHD inattentive symptoms dimension, both for the SNAP-IV (*β* = − 1.75, S.E. = 0.47, *p* < 0.01) and the DSM-IV-TR count (*β* = − 0.57, S.E. = 0.21, *p* = 0.01). For hyperactive–impulsive symptoms, the DSM-IV-TR score was significant (*β* = − 0.42, S.E. = 0.17, *p* = 0.01), but was not significant for SNAP-IV hyperactive–impulsive symptoms (*β* = − 0.77, S.E. = 0.44, *p* = 0.08) (Table 3).

*Age- and sex-matched sensitivity analyses*: As shown in Table 4 (Appendix 1), when we ran the generalised linear models in age- and sex-matched subgroups, an increase in 25(OH)D was no longer associated with higher odds of having CTD (*p* = 0.46), but remained significantly associated with higher odds of having comorbid OCD (OR 1.83, *p* = 0.01) and with lower odds of having comorbid ADHD (OR 0.55, *p* = 0.02) within the CTD cohort. (Note matching the cohorts reduced the sample size and, therefore, the power to detect significant results.)

*25(OH)D sufficiency/insufficiency as a binary predictor variable:* As shown in Tables 5 and 6 (Appendix 1), 25(OH)D sufficiency (> 20 ng/ml) was associated with higher odds of having CTD compared to the unaffected cohort (OR 3.05, *p* = 0.01). Whereas, 25(OH)D sufficiency was not associated with a change in the odds of having OCD within the CTD group (*p* > 0.05). However, 25(OH)D sufficiency was associated with lower odds of having comorbid ADHD (OR 0.54, *p* = 0.05) and a decrease in ADHD symptom severity on total SNAP (*β* = − 4.21, S.E. = 1.49, *p* = 0.01) and DSM-IV-TR (− 1.25, *p* = 0.04), as well as inattentive domains, though not the hyperactivity/impulsivity (*p* > 0.05) subscales, on both measures.

## Discussion

### Main findings

In this multicentre study of children and adolescents with CTD and first-degree relatives of individuals with CTD, lower levels of 25(OH)D were not associated with a higher presence or severity of tics. Contrary to our hypothesis, first-degree relatives who themselves did not develop tics within the study period (unaffected cohort) had *lower* 25(OH)D levels than participants with CTD. Additionally, there was no significant difference in 25(OH)D status between at-risk individuals who went on to develop tics within the study period (tic onset cohort) compared to those who did not (unaffected cohort). However, serum 25(OH)D was associated with psychiatric comorbidities in CTD: higher levels were associated with lower odds of having comorbid ADHD and inversely associated with ADHD symptom severity. Conversely, higher serum 25(OH)D was associated with higher odds of having comorbid OCD, but was not associated with OCD symptom severity.

Vitamin D deficiency is common in Europe and worldwide [4, 5, 32]. A recent meta-analysis combined data from 18 European studies (for total *n* = 55,844) and gave an overall pooled estimate of vitamin D deficiency (< 12 ng/ml), irrespective of the age, ethnicity, and latitude of the study population, of 13.0% [5]. This is a comparatively larger proportion than observed in any of the cohorts included in this study. This estimate is, therefore, in line with our observation that children with CTD are no more likely to have vitamin D deficiency than the general population. Nonetheless, 6.0% of participants in this study were deficient at the time of serum collection and would require treatment according to the National Institute of Health and Care Excellence (NICE) guidelines (< 10 ng/ml; 25 nmol/l) [31]. It therefore remains important to highlight the risk vitamin D deficiency poses to children and adolescents in Europe, particularly as cost-effective prevention strategies, such as altered diet, vitamin D supplements, and changes in behavior related to sun exposure are so easy to implement.

Higher serum 25(OH)D was associated with higher odds of having CTD compared to the unaffected cohort in this study. However, this association was no longer significant when tested for in an age- and sex-matched subgroup. Matching substantially reduced the size of our population sample, and therefore, loss of power may explain why we no longer observed a significant result. However, it is also possible that the difference in 25(OH)D between the two groups was largely due to differences in age and/or sex. Changes in parent and/or child behavior as a direct result of their diagnosis, i.e., “reverse causation”, is another potential factor. Age, sex, and diagnosis may influence activity, sunlight exposure, diet, and use of supplementation as parents and/or clinicians may be more likely to check and thus attempt to remediate vitamin deficiencies in patients with CTD. Lack of data on vitamin D supplementation usage and sun exposure was a limitation of this study, and should be recorded and controlled for in future studies.

Our findings differ from a case–control study and a follow-up supplementation trial in Chinese children, which reported lower levels of 25(OH)D in children with tics; a negative correlation between tic severity and 25(OH)D levels; and observed improvements in tic severity upon 25(OH)D supplementation [24, 25]. It is possible that ethnicity and genetic susceptibility played a role in these divergent results. Another difference between this study and the two previous ones is that this study focussed on the differences between children with CTD and first-degree relatives. Therefore, factors that influence 25(OH)D levels, such as environment and genetics, were more likely to be shared between cohorts which could have potentially reduced confounding influences on 25(OH)D. The key limitations of the supplementation trial in Chinese children, as highlighted by the authors themselves, were that it was open-label and almost half of the children did not complete treatment or were lost to follow-up [25]. Therefore, the observed improvement in tic severity following 25(OH)D supplementation (*n* = 36) could have been due to other factors influencing participation including increased parental support or placebo. As mentioned above, it is important to emphasise that all children and adolescents deficient or insufficient in 25(OH)D should be given appropriate supplementation. However, our findings suggest more robust evidence should be gathered before further supplementation trials are implemented in children with CTD as resources may be better directed elsewhere.

For comorbid OCD in participants with CTD, we found the opposite result to our hypothesis. We observed a 10 ng/ml increase in 25(OH)D levels was associated with higher odds of having comorbid OCD (OR 1.46, 95% CI 1.04–2.04, *p* = 0.03). However, when we looked at 25(OH)D insufficiency versus sufficiency as a binary predictor variable, 25(OH)D sufficiency was no longer a significant predictor of OCD. Furthermore, 25(OH)D sufficiency was not associated with the severity of comorbid OCD symptoms. Studies investigating 25(OH)D levels in individuals with OCD but without comorbid CTD have so far produced mixed results [43]. Again, differences in parent and/or child behavior in response to diagnoses (“reverse causation”) may explain why we observed increased 25(OH)D levels in our study. Nonetheless, given that there seems to be no association with the severity of OCD symptoms and 25(OH)D levels, it is unlikely that low 25(OH)D levels share a causal relationship with OCD symptoms.

However, a 10 ng/ml increase in 25(OH)D was associated with lower odds of having comorbid ADHD in the CTD cohort (OR 0.55, 95% CI, 0.36–0.84, *p* = 0.01). In addition, we observed that for an increase of 10 ng/ml 25(OH)D, the SNAP-IV ADHD scale decreased by an average of 2.52. A clinically meaningful change in ADHD symptoms is often considered as a 30% reduction [40]. In our cohort, this equates to an average 5.6-point reduction in SNAP-IV (range 0–54) which was associated with a 22.4 ng/ml increase in 25(OH)D. The physiological relevance of this degree of increase is not fully understood; current guidelines on 25(OH)D levels are based on its role in bone metabolism, but less is known about the impact of changes in serum levels in relation to its involvement in immune mechanisms, brain development, and neurotransmission. A recent meta-analysis, which included 10,334 children and adolescents, found that children with ADHD had lower levels of 25(OH)D on average when compared to healthy controls [18]. However, our study is the first to assess 25(OH)D in the context of comorbid ADHD in children with CTD. Comorbid ADHD is common in children with CTD (40–80%) [11]. Evidence of possible genetic, and neurobiological and neurophysiological overlap between CTD and ADHD indicate that the two disorders are highly interrelated [11], and those with both CTD and ADHD have a substantially increased psychiatric and functional burden and a lower quality of life [33]. Therefore, understanding and managing ADHD symptoms in children with CTD is of clinical importance. The results of this study indicate that vitamin D status may be helpful as a marker of ADHD disease activity in children with CTD. While further evidence is required, it is possible that supplementation may prove to be beneficial in a proportion of children with ADHD symptoms; however, the benefits may well be restricted to those who are deficient.

## Limitations

One limitation of this study was that we were unable to compare our findings to a healthy control cohort and that we had to control for age and sex in all our models. The EMTICS cohorts were not set up as a regular case–control study and an assessment of vitamin D status was not included in the original research plan. Nonetheless, 25(OH)D remained significantly associated with the presence and severity of comorbid ADHD when we conducted a sensitivity analysis using age- and sex-matched subgroups.

Another limitation of this study was that serum samples for 25(OH)D analysis were only available for a small number of participants with tic onset (*n* = 31). Moreover, our cross-sectional study is not useful for drawing inferences about the direction of effects between hypovitaminosis D and comorbid ADHD in children with CTD. Prospective longitudinal studies are needed to determine whether lower vitamin D levels predispose individuals with CTD to develop comorbid ADHD; or whether those with comorbid ADHD have lower levels of vitamin D due to differences in behavior, immune activation, metabolism (reverse causation); or the two are indirectly linked via  shared mechanisms. Our study also lacked complementary data on diet, parathyroid hormone levels, calcium levels, dairy intake, daily outdoor activity, sunlight exposure, body mass index, puberty stage, supplementation during infancy, medication, socioeconomic status, and screening for comorbid anxiety and depression, which should be considered in future research.

## Conclusion

To our knowledge, this is the largest study to date to investigate 25(OH)D status in children and adolescents with CTD and the first to evaluate 25(OH)D alongside the onset of tics in an at-risk population. It is also the first to investigate 25(OH)D levels in relation to comorbid ADHD and OCD in CTD. In contrast to earlier observations, lower serum 25(OH)D was not associated with a higher presence or severity of tics. However, our data showed an inverse association between serum 25(OH)D levels and both the presence and severity of comorbid ADHD, and this relationship warrants further investigation.

## Appendix

See Tables 4, 5 and 6.

**Table 4:** Sensitivity analysis with age- and sex-matched subgroups

Effect of 25(OH)D on the presence of CTD, comorbid OCD and ADHD			
Dependent variable^a^	OR	95% CI	*p*
CTD	1.18	0.75–1.86	0.46
Tic onset	2.11	0.85–5.26	0.11
CTD comorbid OCD	1.83	1.20–2.80	0.01*
CTD comorbid ADHD	0.55	0.34–0.91	0.02*

^a^The dependent variable is the presence of CTD, tic onset, OCD or ADHD and the primary predictor variable is adjusted 25(OH)D (1-unit change = 10 ng/ml). Sex, age and comorbidity, other than that being tested, were also entered as covariates. The odds are calculated for 1-unit change (i.e., 10 ng/ml) in our predictor variable

*CTD* chronic tic disorder, *OCD* obsessive–compulsive disorder; *ADHD* attention-deficit hyperactivity disorder

*The significant difference with *p* < 0.05

**Table 5:** Effect of 25(OH)D sufficient versus insufficient on the presence of CTD, comorbid OCD, and ADHD

Dependent variable^a^	OR	95% CI	*p*
CTD	3.05	1.39–6.71	0.01*
Tic onset	2.00	0.70–5.72	0.19
CTD comorbid OCD	1.15	0.63–2.11	0.65
CTD comorbid ADHD	0.54	0.29–1.00	0.05*

^a^The dependent variable is the presence of CTD, tic onset, OCD, or ADHD and the primary predictor variable is a binary measure of 25(OH)D insufficiency/sufficiency, where adjusted 25(OH)D insufficiency (≤ 20 ng/ml) = 0 and sufficiency (> 20 ng/ml) = 1. Thus, the odds ratio is calculated for those sufficient as compared to insufficient, i.e., the odds of having CTD were higher (OR = 3.05) in those with sufficient 25(OH)D compared to those with insufficient levels, whereas the odds of having ADHD were lower (OR = 0.54) in those with sufficient 25(OH)D compared to those with insufficient 25(OH)D. Sex, age,and comorbidity, other than that being tested, were also entered as covariates

*CTD* chronic tic disorder, *OCD* obsessive–compulsive disorder, *ADHD* attention-deficit hyperactivity disorder)

*The significant difference with *p* < 0.05

**Table 6:** Effect of 25(OH)D sufficient versus insufficient on Tic, OCD and ADHD symptom severity

Dependent variable^a^	*β*	SE	95% CI	*p*
Tic Severity				
YGTSS:Total	0.85	0.91	− 0.94 to 2.64	0.35
YGTSS Motor	0.19	0.52	− 0.83 to 1.21	0.71
YGTSS Vocal	0.45	0.62	− 0.77 to 1.66	0.72
CGI	− 0.10	0.11	− 0.32 to 0.11	0.34
OCD				
CYBOCs Total	− 0.39	0.90	− 2.16 to 1.39	0.67
CYBOCs Obsessions	0.02	0.49	− 0.52 to 1.40	0.37
CYBOCs Compulsions	− 0.45	0.62	− 1.67 to 0.77	0.47
ADHD				
DSM-IV-TR Total	− 1.25	0.61	− 2.05 to − 0.05	0.04*
DSM-IV-TR Inattentive	− 0.72	0.36	− 1.43 to − 0.01	0.05*
DSM-IV-TR	− 0.46	0.31	− 1.06 to − 0.15	0.14
Hyperactive/Impulsive				
SNAP Total	− 4.21	1.49	− 7.14 to 1.28	0.01*
SNAP Inattentive	− 3.14	0.80	− 4.73 to − 1.55	< 0.01*
SNAP Hyperactive/Impulsive	− 1.39	0.79	− 2.94 to 0.16	0.08

^a^The dependent variable is the presence of CTD, tic onset, OCD, or ADHD and the primary predictor variable is a binary measure of 25(OH)D insufficiency/sufficiency, where adjusted 25(OH)D insufficiency (≤ 20 ng/ml) = 0 and sufficiency (> 20 ng/ml) = 1. *β* coefficients were calculated for the difference between insufficiency (≤ 20 ng/ml) and sufficiency (> 20 ng/ml), i.e., SNAP-total score was less severe (*β* – 4.21) in those with sufficient compared to those with insufficient 25(OH)D. Sex, age, and comorbidity, other than that being tested, were also entered as covariates

*YGTSS* Yale Global Tic Severity Scale 0, *OCD* obsessive–compulsive disorder, *CY-BOCs* Children’s Yale-Brown Obsessive–Compulsive Scale, *ADHD* attention deficit hyperactivity disorder, *DSM-IV-TR* diagnostic and statistical manual of mental disorders 4th ed., text revision, *SNAP-IV* Swanson, Nolan, and Pelham Questionnaire IV

*The significant difference with *p* < 0.05

## References

[1] Aldred M, Cavanna AE (2015). Tourette syndrome and socioeconomic status. Neurol Sci.

[2] American Psychiatric Association (2000). Diagnostic and statistical manual of mental disorders: DSM-IV-TR.

[3] American Psychiatric Association, E (2013). Diagnostic and statistical manual of mental disorders: DSM-5.

[4] Amrein K, Scherkl M, Hoffmann M, Neuwersch-Sommeregger S, Köstenberger M, Tmava Berisha A, Martucci G, Pilz S, Malle O (2020). Vitamin D deficiency 2.0: an update on the current status worldwide. Eur J Clin Nutr.

[5] Cashman KD, Dowling KG, Škrabáková Z, Gonzalez-Gross M, Valtueña J, de Henauw S, Moreno L, Damsgaard CT, Michaelsen KF, Mølgaard C, Jorde R, Grimnes G, Moschonis G, Mavrogianni C, Manios Y, Thamm M, Mensink GB, Rabenberg M, Busch MA, Cox L, Meadows S, Goldberg G, Prentice A, Dekker JM, Nijpels G, Pilz S, Swart KM, van Schoor NM, Lips P, Eiriksdottir G, Gudnason V, Cotch MF, Koskinen S, Lamberg-Allardt C, Durazo-Arvizu RA, Sempos CT, Kiely M (2016). Vitamin D deficiency in Europe: pandemic?. Am J Clin Nutr.

[6] Çelik G, Taş D, Tahiroğlu A, Avci A, Yüksel B, Çam P (2016). Vitamin D deficiency in obsessive-compulsive disorder patients with pediatric autoimmune neuropsychiatric disorders associated with streptococcal infections: a case control study. Noro Psikiyatr Ars.

[7] Cui X, Pertile R, Liu P, Eyles DW (2015). Vitamin D regulates tyrosine hydroxylase expression: N-cadherin a possible mediator. Neuroscience.

[8] Eapen V, Snedden C, Črnčec R, Pick A, Sachdev P (2016). Tourette syndrome, co-morbidities and quality of life. Aust N Z J Psychiatry.

[9] Eddy CM, Rizzo R, Gulisano M, Agodi A, Barchitta M, Calì P, Robertson MM, Cavanna AE (2011). Quality of life in young people with Tourette syndrome: a controlled study. J Neurol.

[10] Eyles DW, Burne TH, McGrath JJ (2013). Vitamin D, effects on brain development, adult brain function and the links between low levels of vitamin D and neuropsychiatric disease. Front Neuroendocrinol.

[11] Ferreira BR, Pio-Abreu JL, Januário C (2014). Tourette's syndrome and associated disorders: a systematic review. Trends Psychiatry Psychother.

[12] Goodman WK, Price LH, Rasmussen SA, Mazure C, Delgado P, Heninger GR, Charney DS (1989). The yale-brown obsessive compulsive scale. II. Validity. Arch Gen Psychiatry.

[13] Greene DJ, Williams AC, Koller JM, Schlaggar BL, Black KJ, TTAOAN Consortium (2017). Brain structure in pediatric Tourette syndrome. Mol Psychiatry.

[14] Guy W (1976) Clinical Global Impression (CGI). ECDEU assessment manual for psychopharmacology.: U.S. Department of Health, Education, and Welfare, Rockville

[15] Hoekstra PJ, Dietrich A, Edwards MJ, Elamin I, Martino D (2013). Environmental factors in Tourette syndrome. Neurosci Biobehav Rev.

[16] Holick MF, Binkley NC, Bischoff-Ferrari HA, Gordon CM, Hanley DA, Heaney RP, Murad MH, Weaver CM, E SOCIETY (2011). Evaluation, treatment, and prevention of vitamin D deficiency: an Endocrine Society clinical practice guideline. J Clin Endocrinol Metab.

[17] Jalenques I, Galland F, Malet L, Morand D, Legrand G, Auclair C, Hartmann A, Derost P, Durif F (2012). Quality of life in adults with Gilles de la Tourette Syndrome. BMC Psychiatry.

[18] Khoshbakht Y, Bidaki R, Salehi-Abargouei A (2018). Vitamin D status and attention deficit hyperactivity disorder: a systematic review and meta-analysis of observational studies. Adv Nutr.

[19] Knight T, Steeves T, Day L, Lowerison M, Jette N, Pringsheim T (2012). Prevalence of tic disorders: a systematic review and meta-analysis. Pediatr Neurol.

[20] Kocovska E, Gaughran F, Krivoy A, Meier UC (2017). Vitamin-D deficiency as a potential environmental risk factor in multiple sclerosis, schizophrenia, and autism. Front Psychiatry.

[21] Kotsi E, Perrea DN (2019). Vitamin D levels in children and adolescents with attention-deficit hyperactivity disorder (ADHD): a meta-analysis. Atten Defic Hyperact Disord.

[22] Leckman JF, Riddle MA, Hardin MT, Ort SI, Swartz KL, Stevenson J, Cohen DJ (1989). The Yale Global Tic Severity Scale: initial testing of a clinician-rated scale of tic severity. J Am Acad Child Adolesc Psychiatry.

[23] Leckman JF, Bloch MH, Smith ME, Larabi D, Hampson M (2010). Neurobiological substrates of Tourette’s disorder. J Child Adolesc Psychopharmacol.

[24] Li HH, Shan L, Wang B, Du L, Xu ZD, Jia FY (2018). Serum 25-hyroxyvitamin D levels and tic severity in Chinese children with tic disorders. Psychiatry Res.

[25] Li HH, Xu ZD, Wang B, Feng JY, Dong HY, Jia FY (2019). Clinical improvement following vitamin D3 supplementation in children with chronic tic disorders. Neuropsychiatr Dis Treat.

[26] Libuda L, Naaresh R, Ludwig C, Laabs BH, Antel J, Föcker M, Hebebrand J, Hinney A, Peters T (2020). A mendelian randomization study on causal effects of 25(OH)vitamin D levels on attention deficit/hyperactivity disorder. Eur J Nutr.

[27] Lit L, Enstrom A, Sharp FR, Gilbert DL (2009). Age-related gene expression in Tourette syndrome. J Psychiatr Res.

[28] Martino D, Johnson I, Leckman JF (2020). What does immunology have to do with normal brain development and the pathophysiology underlying tourette syndrome and related neuropsychiatric disorders?. Front Neurol.

[29] Mataix-Cols D, Isomura K, Pérez-Vigil A, Chang Z, Rück C, Larsson KJ, Leckman JF, Serlachius E, Larsson H, Lichtenstein P (2015). Familial risks of tourette syndrome and chronic tic disorders. A population-based cohort study. JAMA Psychiatry.

[30] Mowry EM, Krupp LB, Milazzo M, Chabas D, Strober JB, Belman AL, McDonald JC, Oksenberg JR, Bacchetti P, Waubant E (2010). Vitamin D status is associated with relapse rate in pediatric-onset multiple sclerosis. Ann Neurol.

[31] NICE (2018) Vitamin D deficiency in children [Online]. National Institute for Health and Care Excellence. https://cks.nice.org.uk/vitamin-d-deficiency-in-children#!scenario

[32] Palacios C, Gonzalez L (2014). Is vitamin D deficiency a major global public health problem?. J Steroid Biochem Mol Biol.

[33] Poh W, Payne JM, Gulenc A, Efron D (2018). Chronic tic disorders in children with ADHD. Arch Dis Child.

[34] Randolph JJ, Falbe K, Manuel AK, Balloun JL (2014) A Step-by Step Guide to Propensity Score Matching in R. 19

[35] Raudenbush SW, Bryk AS (2002). Hierarchical linear models: applications and data analysis methods.

[36] Robertson MM (2008). The prevalence and epidemiology of Gilles de la Tourette syndrome. Part 1: the epidemiological and prevalence studies. J Psychosom Res.

[37] Scahill L, Riddle MA, McSwiggin-Hardin M, Ort SI, King RA, Goodman WK, Cicchetti D, Leckman JF (1997). Children's Yale-Brown Obsessive Compulsive Scale: reliability and validity. J Am Acad Child Adolesc Psychiatry.

[38] Scharf JM, Yu D, Mathews CA, Neale BM, Stewart SE, Fagerness JA, Evans P, Gamazon E, Edlund CK, Service SK, Tikhomirov A, Osiecki L, Illmann C, Pluzhnikov A, Konkashbaev A, Davis LK, Han B, Crane J, Moorjani P, Crenshaw AT, Parkin MA, Reus VI, Lowe TL, Rangel-Lugo M, Chouinard S, Dion Y, Girard S, Cath DC, Smit JH, King RA, Fernandez TV, Leckman JF, Kidd KK, Kidd JR, Pakstis AJ, State MW, Herrera LD, Romero R, Fournier E, Sandor P, Barr CL, Phan N, Gross-Tsur V, Benarroch F, Pollak Y, Budman CL, Bruun RD, Erenberg G, Naarden AL, Lee PC, Weiss N, Kremeyer B, Berrío GB, Campbell DD, Cardona Silgado JC, Ochoa WC, Mesa Restrepo SC, Muller H, Valencia Duarte AV, Lyon GJ, Leppert M, Morgan J, Weiss R, Grados MA, Anderson K, Davarya S, Singer H, Walkup J, Jankovic J, Tischfield JA, Heiman GA, Gilbert DL, Hoekstra PJ, Robertson MM, Kurlan R, Liu C, Gibbs JR, Singleton A, Hardy J, Strengman E, Ophoff RA, Wagner M, Moessner R, Mirel DB, Posthuma D, Sabatti C, Eskin E, Conti DV, Knowles JA, Ruiz-Linares A, Rouleau GA, Purcell S, Heutink P, Oostra BA, Mcmahon WM, Freimer NB, Cox NJ, Pauls DL, NABE, Consortium & UHBE Database (2013). Genome-wide association study of Tourette’s syndrome. Mol Psychiatry.

[39] Schrag A, Martino D, Apter A, Ball J, Bartolini E, Benaroya-Milshtein N, Buttiglione M, Cardona F, Creti R, Efstratiou A, Gariup M, Georgitsi M, Hedderly T, Heyman I, Margarit I, Mir P, Moll N, Morer A, Müller N, Müller-Vahl K, Münchau A, Orefici G, Plessen KJ, Porcelli C, Paschou P, Rizzo R, Roessner V, Schwarz MJ, Steinberg T, Tagwerker Gloor F, Tarnok Z, Walitza S, Dietrich A, Hoekstra PJ, ECGroup (2019). European Multicentre Tics in Children Studies (EMTICS): protocol for two cohort studies to assess risk factors for tic onset and exacerbation in children and adolescents. Eur Child Adolesc Psychiatry.

[40] Steele M, Jensen PS, Quinn DM (2006). Remission versus response as the goal of therapy in ADHD: a new standard for the field?. Clin Ther.

[41] Swanson JM, Kraemer HC, Hinshaw SP, Arnold LE, Conners CK, Abikoff HB, Clevenger W, Davies M, Elliott GR, Greenhill LL, Hechtman L, Hoza B, Jensen PS, March JS, Newcorn JH, Owens EB, Pelham WE, Schiller E, Severe JB, Simpson S, Vitiello B, Wells K, Wigal T, Wu M (2001). Clinical relevance of the primary findings of the MTA: success rates based on severity of ADHD and ODD symptoms at the end of treatment. J Am Acad Child Adolesc Psychiatry.

[42] Tian Y, Gunther JR, Liao IH, Liu D, Ander BP, Stamova BS, Lit L, Jickling GC, Xu H, Zhan X, Sharp FR (2011). GABA- and acetylcholine-related gene expression in blood correlate with tic severity and microarray evidence for alternative splicing in Tourette syndrome: a pilot study. Brain Res.

[43] Yazici KU, Percinel Yazici I, Ustundag B (2018). Vitamin D levels in children and adolescents with obsessive compulsive disorder. Nord J Psychiatry.

[44] Zilhão NR, Olthof MC, Smit DJ, Cath DC, Ligthart L, Mathews CA, Delucchi K, Boomsma DI, Dolan CV (2017). Heritability of tic disorders: a twin-family study. Psychol Med.

